# Silver-enhanced colloidal gold dip strip immunoassay integrated with smartphone-based colorimetry for sensitive detection of cardiac marker troponin I

**DOI:** 10.1038/s41598-022-24458-1

**Published:** 2022-11-18

**Authors:** Napakporn Poosinuntakul, Theerawut Chanmee, Sureerut Porntadavity, Orawon Chailapakul, Amara Apilux

**Affiliations:** 1 Department of Clinical Chemistry, Faculty of Medical Technology, Mahidol University, 999 Phutthamonthon 4 Road, Salaya, Nakhon Pathom 73170 Thailand; 2 Electrochemistry and Optical Spectroscopy Center of Excellence, Department of Chemistry, Faculty of Science, Chulalongkorn University, Bangkok, 10330 Thailand

**Keywords:** Analytical chemistry, Biochemistry, Nanoscience and technology

## Abstract

Cardiac troponin I (cTnI) is a specific cardiac biomarker for diagnosis of acute myocardial infarction (AMI). A sensitive and simple point-of-care test (POCT) is still required for early detection of AMI. To address this need, we developed a dip strip assay based on sandwich immunoassay coupled with a silver enhancement system. Pre-incubation and silver enhancement were introduced to the dip strip to increase sensitivity. Due to the catalytic reaction of the silver enhancement solution, the red color of AuNPs changed to dark brown as silver ions precipitated and enlarged the AuNPs. The obtained results were easily seen by the naked eye. For quantitative analysis, the color intensity of the results was analyzed using a smartphone with RGB color picker application. The effects of operating parameters (volume of AuNP-Ab conjugate, volume of sample, incubation time, and analysis time) were investigated and optimized. Under optimal conditions, the limit of detection (LOD) by the naked eye was 0.5 ng/mL. The LOD with silver enhancement was 50-fold lower than without. For quantitative analysis using the smartphone, linearity of detection was observed through the range of 0.5–50 ng/mL (R^2^ = 0.9952) and the LOD was 0.12 ng/mL. The developed method was successfully applied to detection of cTnI in serum samples, achieving analytical recoveries and %RSD in the ranges of 96.10–119.17% and 2.91–5.13%, respectively. Additionally, this developed assay was not cross reactive with the potentially interfering serum proteins. These results showed the great potential of this dip strip assay as an alternative POCT for detection of serum cTnI.

## Introduction

Cardiovascular diseases (CVDs) are a group of heart and blood vessel disorders. The number and mortality of CVD patients have increased every year and this trend is expected to continue into the future^1,2^. A blockage of the coronary arteries results in reduced blood flow and insufficient blood supply to the heart muscle, leading to its damage. The damaged cardiac muscle will deteriorate and eventually necrose and die. This type of heart damage is known as a myocardial infarction (MI). When the heart is damaged, cardiac biomarkers such as myoglobin, creatine kinase-myocardial band (CK-MB), cardiac troponin T (cTnT) and cardiac troponin I (cTnI), are released into the blood circulation^3^. Currently, cTnI is regarded as the most specific cardiac biomarker and the gold standard for diagnosis of MI due to its unique cardiac isoform^4,5^. cTnI is a protein located between actin filaments of normal cardiac muscle tissue. After the onset of chest pain, the cardiac troponin fragments are elevated in the blood circulation within 4–9 h, with the predominant form being the cTnC-cTnI complex^6^. Its concentration continues to rise until reaching a peak 12–24 h later and remaining elevated for up to 10–14 days. The magnitude of the cardiac troponin elevation is associated with the severity or size of the cardiac injury^7,8^. Accordingly, measurement of cTnI level is part of a clinical risk assessment which enables early diagnosis, prognosis, and monitoring of acute myocardial infarction (AMI) with a cTnI level of 0.4 ng/mL or greater considered indicative of an AMI^9–11^.

Nowadays, the conventional method for cardiac biomarker measurement is electrochemiluminescence immunoassay (ECLIA)^12–14^. Although the method provides high sensitivity and specificity, it requires a large bench-top detection system and skilled operators. Moreover, the instruments are very expensive and have high operating costs. Alternatively, POCT devices are portable tools which help medical staff deliver immediate care to patients. Lateral flow immunoassays (LFIAs) are potential screening tools used as POCTs. This is because they are cost effective and are simple tests which can be used with minimal training. In recent years, LFIAs have been reported for measurement of cardiac troponin I using various signal labels such as dual gold nanoparticles^15^, fluorescent microspheres^16^, quantum dots^17^, magnetic particles^18–20^, enzyme-catalyzed labeling^21,22^, upconverting nanoparticles^23,24^, and ion-doped nanoparticles^25^. However, these assays have their own limitations. For example, fluorescent particles require a fluorescence reader, and one needs to be concerned about photostability and quantum yield. For enzyme-catalyzed labeling, the reports describe interactions between enzyme and substrate, and their analysis using chemiluminometric detectors. These techniques are complicated by the associated sensor readers, which require high operation and material expenses. Moreover, the processes for signal labeling of antibody are cumbersome.

Colloidal gold nanoparticles (AuNPs) have been widely used as labels with antibodies for colorimetric immunoassays because of their biocompatibility, simplicity of synthesis, stability, precision, and high performance optical properties^26^. However, they are normally limited by a low sensitivity of detection. Several groups have employed signal amplification, including silver enhancement, to improve sensitivity of these AuNP-based immunoassays^27–29^. This method uses catalytic silver ion reduction and the AuNPs act as cores, leading to silver ion precipitation on AuNP surfaces. The large particle size of the AuNPs provide the dark color and even increase their intensity. This makes them more visible, allowing results to be read out by naked eyes. A poly(dimethylsiloxane) (PDMS) chip with silver enhancement has been proposed for colorimetric detection of cTnI^28^. However, the fabrication of such a chip is a complicated procedure and the PDMS is easily deformable, sometimes leading to imprecise pattern placement. Additionally, the immunogold silver staining to enhance sensitivity for cTnI detection uses 96-well plates, a process which requires trained staff and is time consuming^29^. In this work, the silver enhancement method was introduced for increasing the sensitivity in the dip strip assay.

Recently, POCT devices have been integrated with colorimetric assay for simple and rapid detection^30,31^. Their results can be easily interpreted using a digital camera with an image processing feature to quantify the color intensity of the results related to the concentration of analyte. Nowadays, smartphones equipped with digital cameras have begun to be employed as a data analysis tools. With appropriate software, they have been used for detection and quantification for colorimetric measurements. Some smartphones are established as portable analytical detectors due to their simplicity, portability and low cost when compared with the commercial instruments^32^. Therefore, the use of a smartphone is an alternative choice for quantitative measurement.

In this study, a AuNP-based dip strip utilizing a sandwich immunoassay coupled with silver enhancement was developed for use as a POCT device, providing a simple and sensitive assay that eliminates the need for complicated and expensive equipment. By this assay, positive results were dark brown in color and could be easily observed by the naked eye. The sensitivity of detection was improved 50-fold when compared with the test without silver enhancement. Also, smartphone-based colorimetry was integrated into the system for simple quantitative measurement of cTnI. The developed device was successfully applied for cTnI determination in serum samples with good accuracy and reliability.

## Results and discussion

### Optimization and characterization of anti-cardiac troponin I antibody-conjugated AuNPs

**Figure 1:**
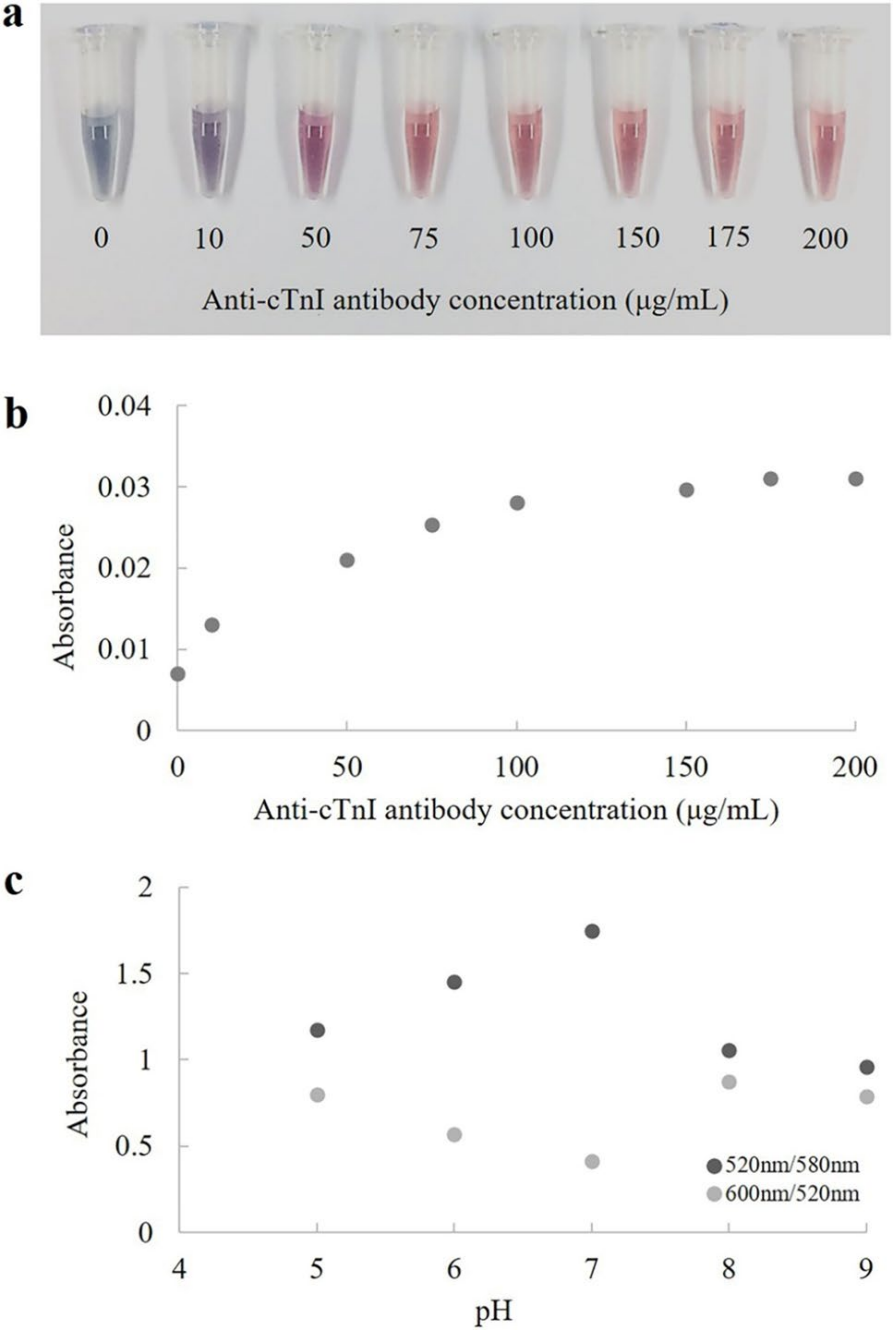
(**a**) The results of AuNP solutions with different concentrations of anti-cTnI antibody after adding 10% NaCl. (**b**) Plot of absorbance observed at 520 nm versus concentration of anti- cTnI antibody. (**c**) Plot of absorbance ratios of AuNPs across pH after adding of 10% NaCl with a fixed anti-cTnI antibody concentration of 175 µg/mL.

To conjugate AuNPs with antibody, the effects of concentration of anti-cardiac troponin I (anti-cTnI) antibody and pH of AuNPs solution were investigated. The optimal concentration of anti-cTnI antibody for conjugation with AuNPs was determined based on results with a series of concentrations (0, 10, 50, 75, 100, 150, 175 and 200 µg/mL). Subsequently, 10% NaCl was added into the mixture containing anti-cTnI antibody and AuNPs. The results are shown in Fig. 1a. Upon the addition of NaCl, AuNP aggregation was induced resulting in a color change of AuNPs from red to blue. At low concentrations of anti-cTnI antibody, the color of AuNPs was violet or red-violet due to excess free AuNPs. Whereas, there was no color change of AuNP suspensions at the high concentrations of anti-cTnI antibody due to sufficient antibody binding. The UV–visible spectra of AuNPs (Fig. 1b) showed that the absorbance (λ_max_ = 520 nm) increased with increasing anti-cTnI antibody concentration and reached a plateau at 175 µg/mL. Therefore, 175 µg/mL anti-cTnI antibody was selected as the concentration to be used in the next experiment.

Since pH affects AuNP-Ab binding activity and stability, it was studied by adjusting the pH values from 5 to 9. The stability and polydispersity of the colloidal solution were determined by calculating the absorbance ratio at the λ_max_/580 nm and 600 nm/λ_max_ (λ_max_ = 520 nm), respectively^27^. The results (Fig. 1c) showed that the most stability and least polydispersion occurred at pH 7. Therefore, this optimal pH was chosen for subsequent preparation of anti-cTnI antibody-conjugated AuNPs.

**Figure 2:**
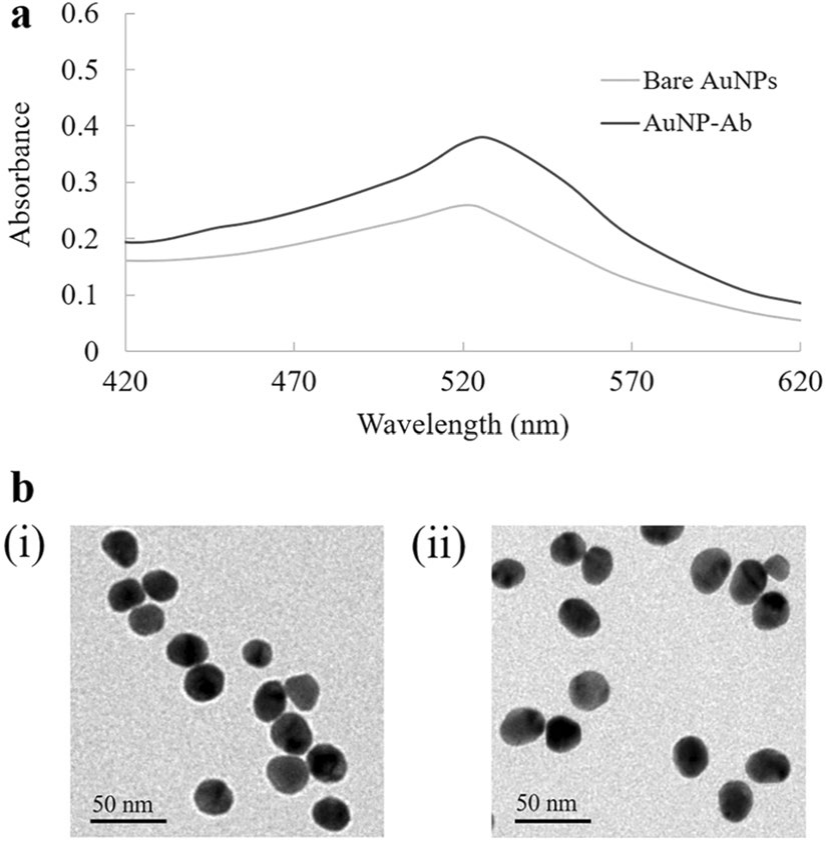
(**a**) Surface plasmon extinction spectra (by UV–visible spectra) of bare and Ab-conjugated AuNPs. (**b**) Transmission electron microscopic images of (i) AuNPs and (ii) Ab-conjugated AuNPs.

To characterize the anti-cTnI antibody-conjugated AuNPs, UV–visible spectra and TEM images were used to compare bare and Ab-conjugated AuNPs. As shown in Fig. 2a, unconjugated AuNPs possessed a surface plasmon extinction spectrum with a maximum peak at 520 nm. After conjugation of AuNPs with anti-cTnI antibody, the UV–vis spectra showed a slightly increased absorption spectra with the peak slightly shifted (to 530 nm). This spectral shift was caused by a change in the localized surface plasmon resonance affected by the protein adhesion layer. Additionally, TEM images (Fig. 2b) showed that the average diameter of AuNPs was 20 nm. After physical absorption of antibody on AuNPs, the average diameter increased to 25 nm. These results indicated successful conjugation of anti-cTnI antibody on the AuNPs^33,34^.

### Signal enhancement of the dip strip immunoassay

**Figure 3:**
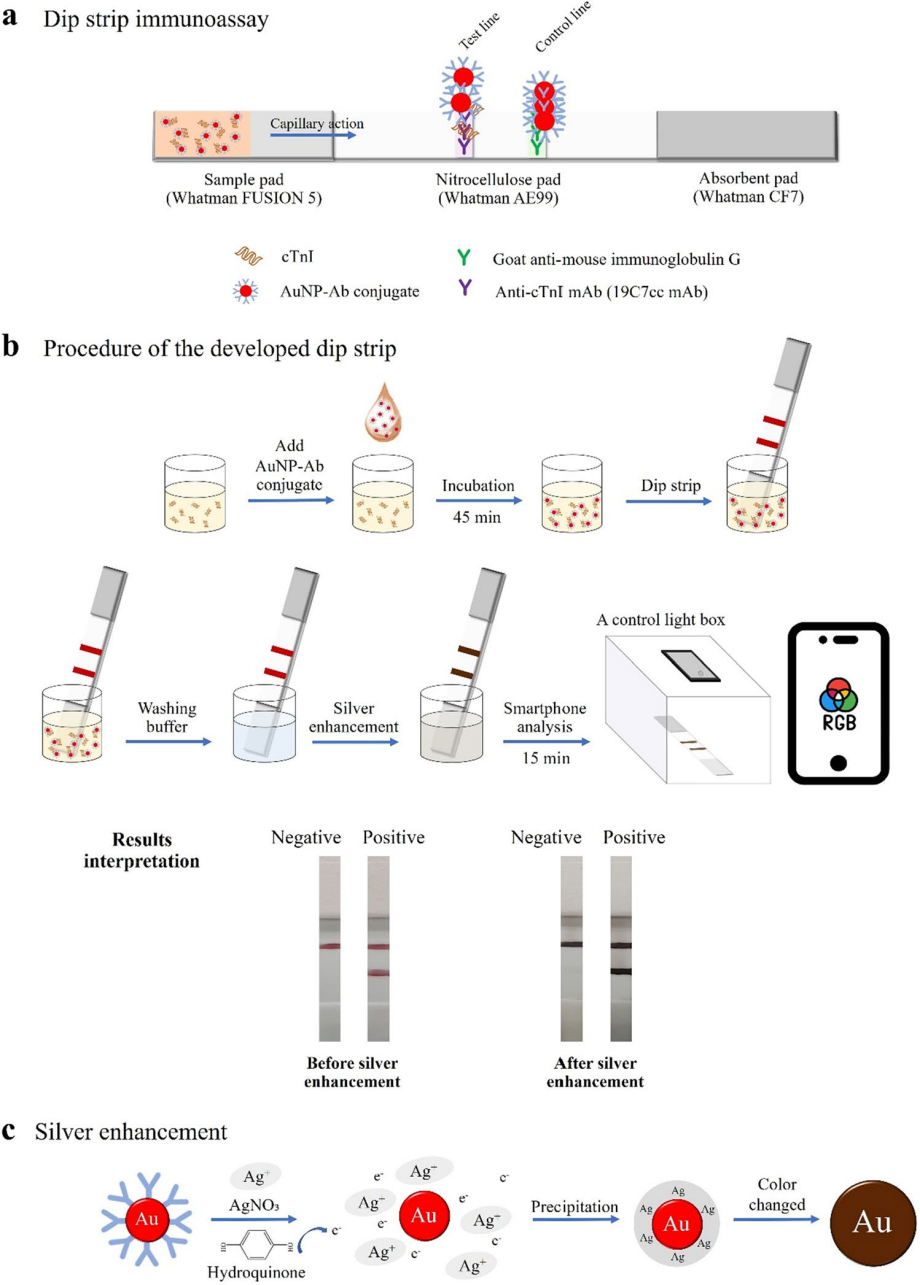
(**a**) Schematic illustration of the dip strip based on sandwich immunoassay. (**b**) Schematic diagram of the dip strip procedure. (**c**) Schematic of silver enhancement reaction showing the change of AuNP color.

To obtain the highly sensitive detection of cTnI needed for diagnosis of AMI, a dip strip based on sandwich immunoassay with pre-incubation and signal enhancement steps was developed, coupled with a smartphone-based colorimetric detection application (Fig. 3b). Pre-incubation of AuNP-Ab conjugate with sample was introduced in this developed assay to complete the binding of the AuNP-Ab conjugate with cTnI. This procedure is shown to increase the sensitivity, reproducibility, and accuracy of the test^35^. After placing the dip strip into the mixture of AuNP-Ab conjugate and sample containing cTnI, the cTnI-AuNP-Ab conjugate migrated through a nitrocellulose membrane by capillary action.

In the presence of cTnI, immobilized anti-cTnI antibody specifically bound the cTnI-AuNP-Ab conjugate at the test line. Meanwhile, excess free AuNP-Ab conjugate flowed to the control line and reacted with the goat anti-mouse antibody. The results were seen as a red color at both the test and control lines. On the other hand, in the absence of cTnI, the results showed the red color only at the control line. However, detection with this method had low sensitivity.

To increase the sensitivity, silver enhancement was applied to the dip strip based on a chromogenic reaction catalyzed by AuNPs (Fig. 3c). After the mixture of silver nitrate and hydroquinone solution was applied to the dip strip, the silver ions were reduced by hydroquinone to form a shell on AuNPs. The red color of AuNPs changed to a dark brown color apparently due to the silver ions precipitated on the AuNP surfaces. Positive results (in the presence of cTnI) exhibited a dark brown color at both the test and control lines, while negative results (in the absence of cTnI) indicated the dark brown color only at the control line. Test results were detectable and could be interpreted semi-quantitatively by the naked eye. Moreover, the results could be quantitated using RGB intensity by smartphone with an RGB application.

### Optimization parameters of the dip strip immunoassay

Parameters likely to influence the sensitivity of the dip strip were investigated. These included volume of anti-cTnI antibody-conjugated AuNPs, sample volume, incubation time, and analysis time. To obtain results, the RGB intensity at the test line after testing with 250 ng/mL of cTnI followed by signal enhancement were interpreted by smartphone with an RGB color picker application.

**Figure 4:**
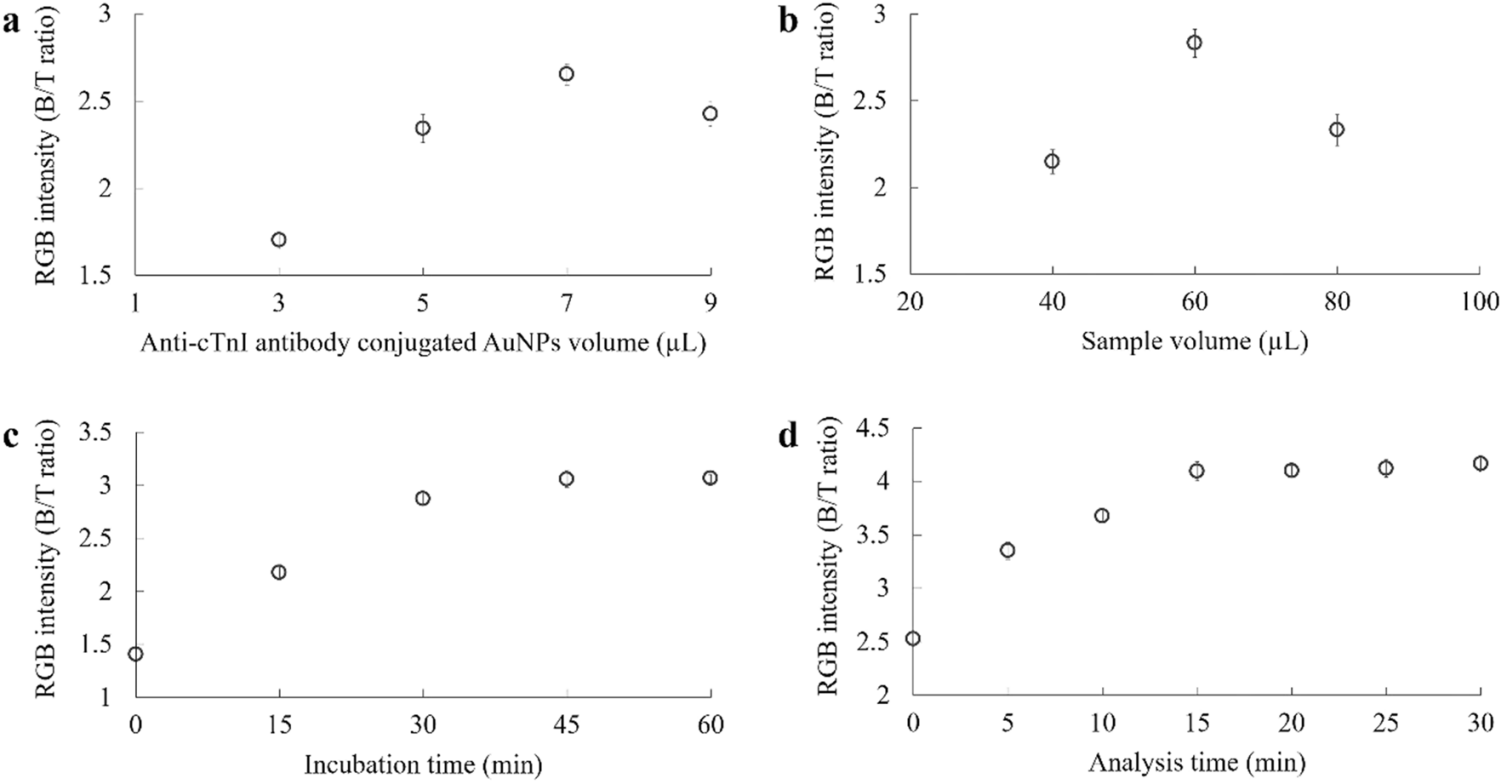
Optimization of various parameters which affect the dip strip immunoassay: (**a**) AuNP-Ab conjugate volume, (**b**) sample volume, (**c**) incubation time, and (**d**) analysis time. Data points represent the mean of three replicates (n = 3), error bar.

The effect of AuNP-Ab conjugate volume was optimized by adding AuNP-Ab conjugate in volumes of 3, 5, 7 or 9 µL to samples containing cTnI at 40 µL with subsequent incubation for 60 min. The highest RGB intensity (B/T ratio) was obtained when the volume of the AuNP-Ab conjugate was 7 µL (Fig. 4a). Therefore, this concentration of AuNP-Ab conjugate was used for further experiments.

Sample volumes (40, 60 and 80 µL) were comparatively evaluated. As shown in Fig. 4b, color intensity increased with increasing in sample volume from 40 to 60 µL. The highest RGB intensity (B/T ratio) at the test line was exhibited at 60 µL. Then the color intensity decreased at 80 µL. The ‘hook effect’ occurred when the sample volume was over 60 µL due to high concentration of analyte. Based on these results, 60 µL was selected as the optimal sample volume for the test strip.

The effect of incubation time on antigen–antibody binding was examined at room temperature for 0, 15, 30, 45 and 60 min. As shown in Fig. 4c, the B/T of RGB intensity at the test line increased as the incubation time was increased and reached a plateau state at 45 min. This indicated that the reaction between AuNP-Ab conjugate and cTnI was complete. We thus chose 45 min as the optimal incubation time for the developed assay, a shorter time than required by the commercial ELISA kit.

One of the most significant characteristics of a diagnostic assay is the analysis time. In this assay, the silver enhancer caused the color at test line to change from red to dark brown. The analysis time for result interpretation was investigated over 30 min (at 0, 5, 10, 15, 20, 25 and 30 min). The color intensity increased with the increasing of analysis time from 0 to 15 min (Fig. 4d). The highest B/T of RGB intensity at the test line was reached at 15 min, and did not increase further. The test results on the strip were stable at 15–30 min. Therefore, 15 min was chosen for the optimal analysis time for the test strip procedure.

### Analytical performance of the developed dip strip immunoassay for cTnI determination

**Figure 5:**
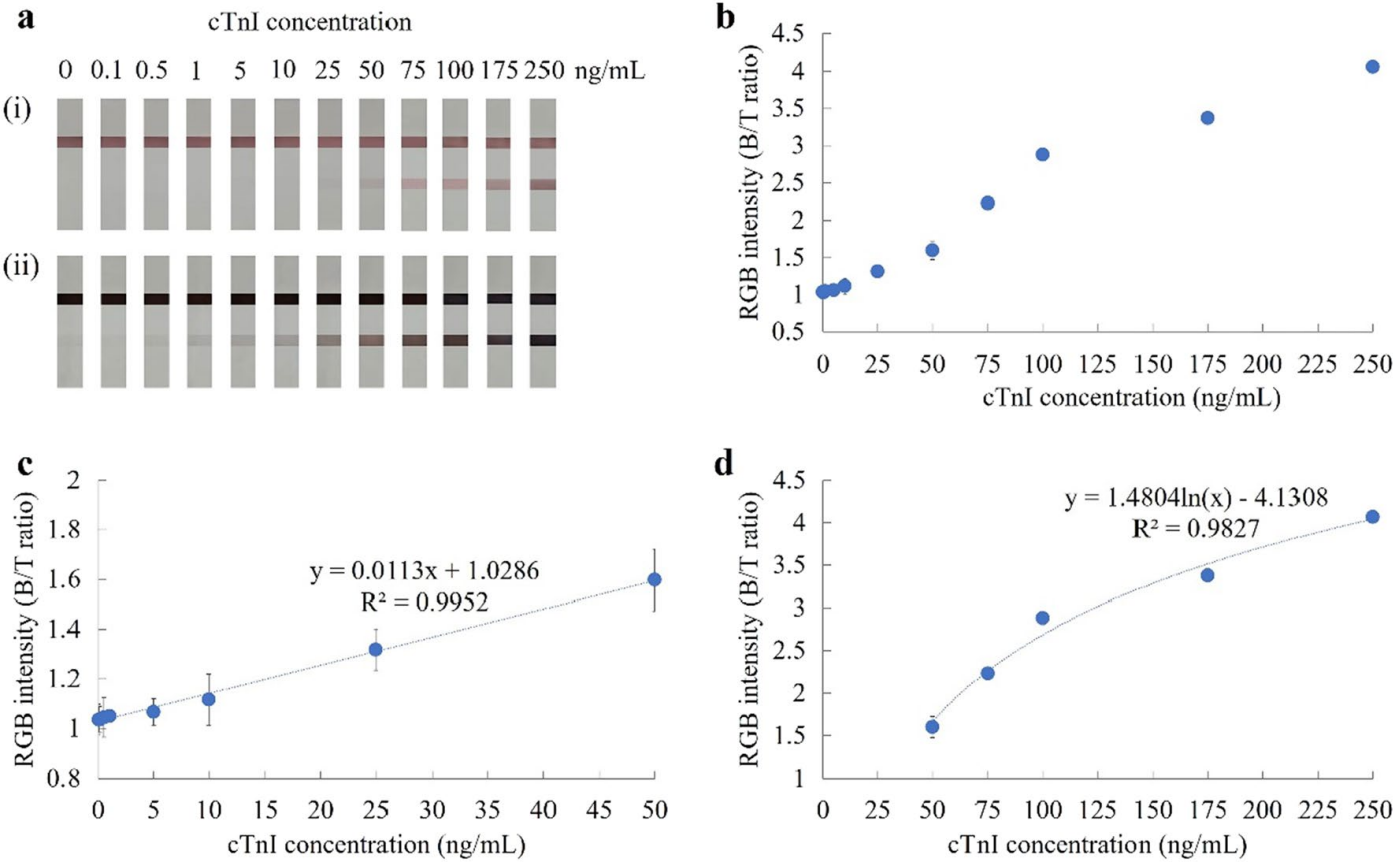
(**a**) Images of cTnI detected across concentrations of 0–250 ng/mL (i) before, and (ii) after silver enhancement. (**b**) Plot of RGB intensity (B/T ratio) versus concentration of cTnI as interpreted by smartphone with RGB color picker application. (**c**) Calibration curve of cTnI concentration in the range of 0–50 ng/mL. (**d**) Calibration curve of cTnI concentration in the range of 50–250 ng/mL.

Performance of the developed dip strip was analyzed under the optimal conditions described above. Serum samples containing cTnI were analyzed by electrochemiluminescence to determine cTnI concentration. Results were obtained using sera spiked with cTnI at concentrations ranging from 0 to 250 ng/mL (Fig. 5a). Color intensity at the test line was monitored before and after signal enhancement. The limit of detection (LOD) achieved by naked eye without and with silver enhancement was 25 and 0.5 ng/mL, respectively. Thus, silver enhancement produced a 50-fold increase in the sensitivity of this developed cTnI test strip.

For quantitative measurement using smartphone, the outcome of interest was the RGB intensity (B/T ratio) at the test line which was obtained using an RGB color picker application. RGB intensity against cTnI concentration was plotted (Fig. 5b). The B/T ratios of RGB intensity were found to increase proportionally with cTnI concentration. However, when cTnI concentration reached 100 ng/mL, the increase in intensity slowed and appeared to stabilize at 250 ng/mL. As shown in Fig. 5c, there was a good linear relationship between intensity of signal and cTnI concentration in the range of 0.5–50 ng/mL. The linear equation and its corresponding correlation coefficient were Y(Linear) = 0.0113x + 1.0286 and R^2^ = 0.9952, respectively, where Y is the RGB intensity (B/T ratio) at the test line and X is the cTnI concentration. The error bars of the RSD are derived from three independent measurements. The calculated LOD (3.3SD/slope) was found to be 0.12 ng/mL, a value lower than the cTnI cutoff level (0.4 ng/mL) used for diagnosis of acute myocardial infarction (AMI)^9–11^. Moreover, higher cTnI concentrations (50–250 ng/mL) could be obtained from the calibration plot as a function of logarithmic trendline (Fig. 5d). The logarithmic equation and its corresponding correlation coefficient were Y(Logarithmic) = 1.4804ln(X) − 4.1308 and R^2^ = 0.9827, respectively. Since with onset of chest pain, the level of serum cTnI rises within 4 h, peaks in 12–24 h, and remains elevated up to 10–14 days^36^, our developed dip strip assay could potentially be used for diagnosis of AMI.

### Cross-reactivity study

**Figure 6:**
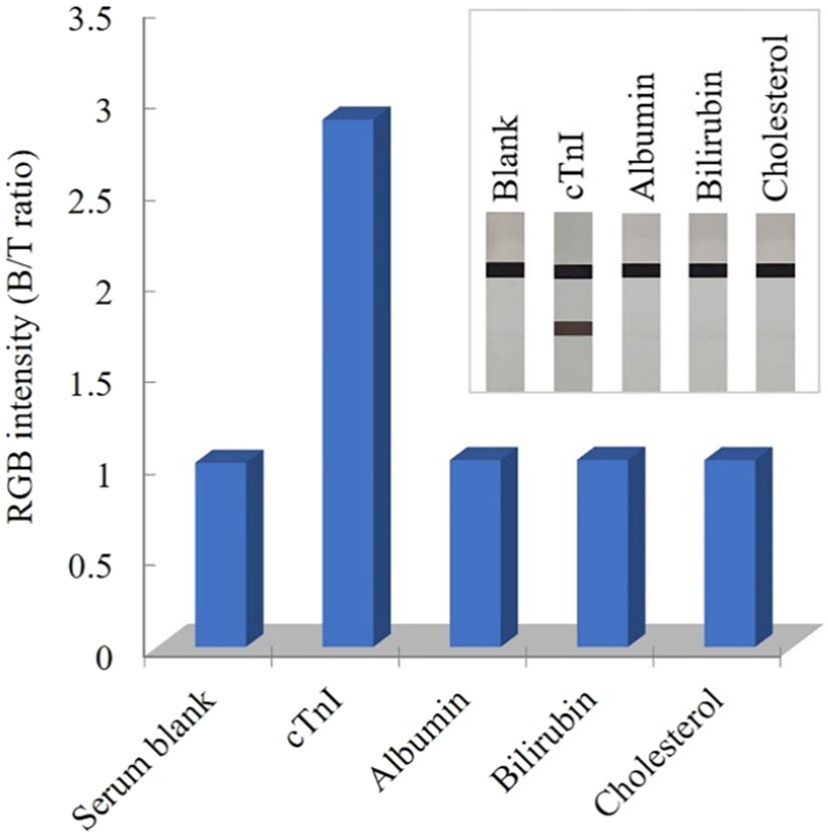
Bar graph and images of cross-reactivity test after silver enhancement, assessing color intensity of B/T ratio of serum blank, cTnI, albumin, bilirubin and cholesterol.

Possible cross-reactivity of the developed assay was investigated to evaluate its specificity in the presence of potentially interfering substances found in serum. Tests were performed with albumin, bilirubin and cholesterol at levels within their normal ranges for human serum (4000 mg/dL albumin, 1 mg/dL bilirubin, and 200 mg/dL cholesterol). As shown in Fig. 6, the test results demonstrated that the dark brown color appeared at the test line in the presence of 100 ng/mL cTnI, but not with any of the other tested substances. Furthermore, there are no significant differences in the color intensity of the test line of interference containing samples compared with the serum blank. These results indicated that the developed dip strip immunoassay retained its specificity toward cTnI determination despite presence of albumin, bilirubin and cholesterol. For interfering substance, hemoglobin may cause interference if the hemolyzed serum was presented^37,38^. The results of cardiac troponin I concentration should therefore be interpreted with caution for mildly hemolyzed serum with the slightly detectable pink color, while the moderately or severely hemolyzed serum should be rejected.

### cTnI determination in human sera

In order to validate an accuracy and precision of the developed dip strip immunoassay for cTnI determination, the developed test strip was used to determine cTnI in serum sample. Sera were spiked with cTnI protein to designated concentrations of 3, 11, 27 and 80 ng/mL. Results are shown in Table 1. Analytical recoveries and %RSD were achieved in the ranges of 96.10–119.17% and 2.91–5.13%, respectively. This developed dip strip immunoassay thus provided accurate and precise detection of cTnI in serum samples.

**Table 1.** Application of the developed dip strip immunoassay for detection of cardiac troponin I in serum samples.

	Spiked cTnI (ng/mL)	Measured cTnI (ng/mL)	% Recovery (n = 3)	% RSD
Serum no. 1	0	ND	–	–
Serum no. 2	3	3.58	119.17	5.13
Serum no. 3	11	10.96	99.65	3.91
Serum no. 4	27	25.95	96.10	2.91
Serum no. 5	80	83.68	104.6	3.22

All data were a mean of three replicated measurements (n = 3).

ND, not detectable.

## Conclusions

A simple and sensitive dip strip-based sandwich immunoassay, coupled with silver-enhanced colloidal gold, was developed for detection and quantitation of cTnI. A silver enhancement system was employed for signal amplification in AuNPs of the developed dip strip. The results were easily observed by naked eye (LOD 0.5 ng/mL). Moreover, a smartphone with RGB color picker application was evaluated because of its simplicity, low cost, and innovative approach for quantitation of results. Using interpretations based on RGB intensity, the LOD was found to be 0.12 ng/mL with a good linear correlation (R^2^ = 0.9952) in the range of 0.5 to 50 ng/mL. The developed assay exhibited encouraging potential for cTnI detection in human serum. It showed outstanding specificity for cTnI detection with no cross-reactivity with important plasma proteins. Consequently, this approach to an alternative assay for analysis of cTnI in patients with suspected AMI is very promising.

## Materials and methods

### Reagents and materials

Goat anti-mouse immunoglobulin G was obtained from Jackson ImmunoResearch Laboratories, Inc. (West Grove, PA, USA). Cardiac troponin I monoclonal antibody (4T21cc–19C7cc) and cardiac troponin I monoclonal antibody (4T21cc–560) were purchased from HyTest Ltd. (Turku, Finland). Cardiac troponin I-C complex was obtained from MyBioSource, Inc. (San Diego, CA, USA). Albumin, bilirubin, cholesterol, gold (III) chloride trihydrate (HAuCl_4_•3H_2_O), human serum, phosphate buffered saline (PBS), silver nitrate and Tween 20 were purchased from Sigma-Aldrich (St. Louis, MO, USA). Bovine serum albumin (BSA) was purchased from Capricorn Scientific GmbH (Ebsdorfergrund, Germany). Hydroquinone was purchased from Tokyo Chemical Industry Co., Ltd. (Tokyo, Japan). Citric acid monohydrate and sodium citrate dihydrate were obtained from Bio Basic Inc. (Ontario, Canada). All analytical-grade chemicals and reagents, and mill-Q (18 MΩ-cm) water were used in the experiments.

### Synthesis of AuNPs

The AuNPs (20 nm) were synthesized using a slight modification of a procedure previously reported^39^. In brief, 100 mL of 0.01% HAuCl_4_ dissolved in milli-Q water was boiled with continuous stirring. Two mL of 1% trisodium citrate was added and stirred for 5 min (until the red color appeared), then the solution was cooled at room temperature with continuous stirring. Finally, 20 nm of AuNPs were obtained. The AuNP solution was stored at 4 °C in a foil-covered bottle for further use. The synthesized AuNPs were characterized by UV–visible spectrometry using a NanoDrop 2000 Spectrophotometer (Thermo Fisher Scientific, Waltham, MA, USA) and transmission electron microscopy (TEM) using a JEM-2100 Electron Microscope (JEOL Ltd., Tokyo, Japan) as shown in Fig. 2b (i).

### Preparation and characterization of anti-cTnI antibody-conjugated AuNPs

The anti-cTnI antibody-conjugated AuNPs were prepared by mixing of 100 µL of 175 µg/mL anti-cTnI antibody and 1 mL of 20 nm AuNPs at pH 7, followed by incubation for 1 h at room temperature with continuous stirring. Thereafter, 100 µL of 5% BSA in 10 mM PBS pH 7.4 was added to the solution to reduce nonspecific binding of AuNPs with other molecules. After incubation for 1 h at room temperature, the mixture was centrifuged at 12,000 rpm for 45 min at 4 °C, with subsequent removal of the supernatant. The pellet was then resuspended in 5% BSA in 10 mM PBS pH 7.4 to a final volume of 100 µL. This ready-to-use anti-cTnI antibody-conjugated AuNP solution was stored at 4 °C. Anti-cTnI antibody-conjugated AuNPs were characterized by NanoDrop 2000 Spectrophotometer (Thermo Fisher Scientific, Waltham, MA, USA) and JEM-2100 Electron Microscope (JEOL Ltd., Tokyo, Japan) as shown in Fig. 2b (ii).

### Silver enhancement

To increase the sensitivity of detection, a silver enhancement method was utilized in this study. The silver enhancement solution was freshly prepared by mixing 2 mg/mL silver nitrate with 5 mg/mL hydroquinone in citrate buffer pH 3.8 in a 1:1 (V:V) reagent ratio.

### Fabrication the dip strip immunoassay

The developed test strip was composed of 3 parts, including a sample pad (Whatman FUSION 5), nitrocellulose pad (Whatman AE99), and absorbent pad (Whatman CF7) which were sequentially stuck on a plastic backing card (GE Healthcare, Pittsburgh, PA, USA) as shown in Fig. 3a. In order to prepare the test strip for cTnI detection, 1 µL of 0.5 mg/mL goat anti-mouse immunoglobulin G and 1 µL of 1 mg/mL of anti-cTnI antibody were immobilized on the nitrocellulose pad at control and test lines, respectively. Finally, the prepared test strip was dried for 2 h. (The fabricated strip had a width of 4 mm.) The ready-to-use test strip was sealed and stored at 4 °C. In addition, the test strip was stable for a storage period of at least 3 months.

### Analysis of cTnI using a dip strip immunoassay observed by naked eyes and smartphone

The test procedure of a dip strip (based on sandwich immunoassay) for cTnI determination is shown in Fig. 3b. Sixty µL of serum sample or cTnI standard was mixed with 7 µL antibody-conjugated AuNPs (AuNP-Ab conjugate) and incubated for 45 min in the sample well. Next, the test strip was dipped into the well. After 5 min, 80 µL of a running buffer (0.1% Tween 20 in 10 mM PBS pH 7.4) was added to reduce background noise. Subsequently, 80 µL of the silver enhancement solution was added to complete the procedure. The test result was interpreted after 15 min by naked eye and smartphone detection. A negative result was indicated by the presence of only a red or dark brown control line if before and after enhancement, respectively. In the case of a positive result, both test and control lines appeared, red/pink and dark brown, before and after enhancement, respectively. The intensity of the color at the test line varied directly with the concentration of cTnI. The concentration of the sample could be estimated by eye in comparison with the cTnI standard. For quantitative analysis, an image of test lines was taken with a smartphone (Samsung S10) with automatic mode and no flash under a control light box. Mean red–green–blue (RGB) intensity values were analyzed using smartphone with an RGB color picker application. Then, the intensity of background per test (B/T) ratio was calculated using the equation: I (intensity) = (R + G + B)/3. The obtained value was used to obtain the concentration of cTnI with a calibration curve.

## Data Availability

The datasets used and/or analysed during the current study available from the corresponding author on reasonable request.
